# Implication of the double-gating mode in a hybrid photon counting detector for measurements of transient heat conduction in GaAs/AlAs superlattice structures

**DOI:** 10.1107/S1600576723004302

**Published:** 2023-06-16

**Authors:** Denys Naumenko, Max Burian, Benedetta Marmiroli, Richard Haider, Andrea Radeticchio, Lucas Wagner, Luca Piazza, Lisa Glatt, Stefan Brandstetter, Simone Dal Zilio, Giorgio Biasiol, Heinz Amenitsch

**Affiliations:** a Institute of Inorganic Chemistry, Graz University of Technology, Stremayrgasse 9/IV, Graz 8010, Austria; b DECTRIS Ltd, Taefernweg 1, Baden-Daettwil 5405, Switzerland; c CNR-IOM – Istituto Officina dei Materiali, SS 14, km 163.5, Basovizza (Trieste) 34149, Italy; Argonne National Laboratory, USA

**Keywords:** pump–probe X-ray diffraction, non-Fourier heat transport, GaAs/AlAs superlattices, hybrid photon counting detectors

## Abstract

Understanding and control of thermal transport in solids at the nanoscale are crucial in engineering and enhance the properties of a new generation of optoelectronic, thermoelectric and photonic devices. In this regard, semiconductor superlattice structures provide a unique platform to study phenomena associated with phonon propagations in solids such as heat conduction. Transient X-ray diffraction can directly probe atomic motions and therefore is among the rare techniques sensitive to phonon dynamics in condensed matter. Here, optically induced transient heat conduction in GaAs/AlAs superlattice structures is studied using the EIGER2 detector. Benchmark experiments have been performed at the Austrian SAXS beamline at Elettra–Sincrotrone Trieste operated in the hybrid filling mode. This work demonstrates that drifts of experimental conditions, such as synchrotron beam fluctuations, become less essential when utilizing the EIGER2 double-gating mode which results in a faster acquisition of high-quality data and facilitates data analysis and data interpretation.

## Abstract 2

Use of the double-gating mode implemented on the modern hybrid photon counting system EIGER2 helps to suppress the influence of beam fluctuations in pump–probe experiments at synchrotron radiation facilities and provides better data quality.

## 1. Introduction

Many fundamental physical, chemical, biochemical *etc*. processes in nature involve structural changes of matter on femtosecond to picosecond timescales that are the natural oscillation periods of atoms and molecules (Siders *et al.*, 1999 ▸; Ihee *et al.*, 2010 ▸). Visible lasers with femtosecond/picosecond pulse widths are intensively used to optically pump and dynamically probe a wide range of atomic, molecular, solid-state and plasma systems (Galli *et al.*, 1990 ▸; Ruello & Gusev, 2015 ▸; Fischer *et al.*, 2016 ▸; Basiri *et al.*, 2022 ▸). The drawback of all optical pump–probe techniques is that visible light cannot resolve atomic scale features since it does not interact with the core electrons and nuclei that most directly indicate a structure. Nevertheless, it interacts with valence and free electrons and probes the ensuing dynamics inherently (Chergui & Collet, 2017 ▸; Bencivenga *et al.*, 2019 ▸; Maiuri *et al.*, 2020 ▸). Hard X-ray radiation, with wavelengths comparable to inter­atomic distances, is well suited to investigate structure and atomic rearrangement and can measure structural dynamics in the interior of samples which are not transparent to ordinary light (Rose-Petruck *et al.*, 1999 ▸; Lindenberg *et al.*, 2017 ▸). Recently, high-brightness free-electron lasers and synchrotron sources have been used in new classes of visible-pump/X-ray-probe experiments to follow structural changes in solid-state systems, and organic and protein crystals with excellent spatial resolution (March *et al.*, 2011 ▸; Chergui & Collet, 2017 ▸; Bencivenga *et al.*, 2019 ▸; Maiuri *et al.*, 2020 ▸). In the past few years, the Austrian SAXS beamline at Elettra–Sincrotrone Trieste has implemented such a pump–probe X-ray diffraction/scattering setup and demonstrated that light-induced structural changes in semiconductor nanostructures and metal–organic frameworks on timescales ranging from picoseconds to seconds, respectively (Burian *et al.*, 2020 ▸; Klokic *et al.*, 2022 ▸), can be successfully studied. Nevertheless, choosing the right data acquisition system in pump–probe experiments is always a challenge mainly because of intensity fluctuations and long-term stability of light sources, beamline optics and sample environment. In this work, an implementation of double-gating mode of a hybrid photon counting detector allows a better correction for instabilities of experimental conditions. We demonstrate this by decoupling complex phenomena at sub-nanosecond timescales in optically excited GaAs/AlAs superlattice (SL) structures.

## 2. Experimental setup and methodology

**Figure 1:**
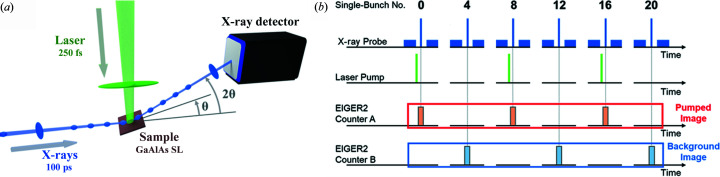
(*a*) Sketch of the experimental setup and (*b*) synchronization scheme of laser pump and X-ray probe to the storage ring filling pattern. Every fourth single bunch is displayed in panel (*b*). A more detailed filling pattern is shown in Fig. S1.

**Figure 2:**
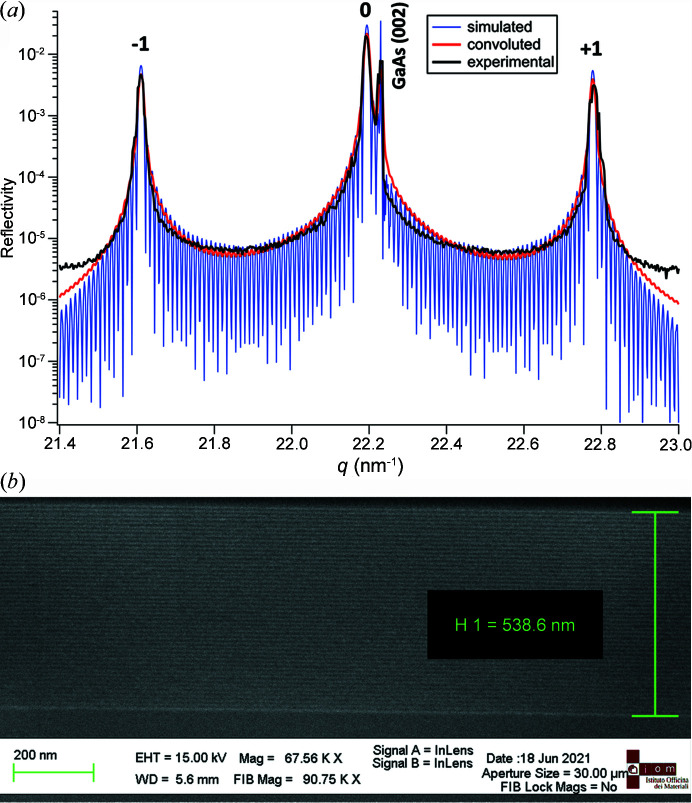
(*a*) Simulated rocking curve of GaAs/AlAs SL (blue), its convolution with the instrumental function (red) and the experimental data (black). (*b*) Cross-sectional SEM image of the corresponding SL with measured total SL thickness (tilt corrected).

All experiments have been performed at the Austrian SAXS beamline (Amenitsch *et al.*, 1998 ▸) at Elettra–Sincrotrone Trieste operated in the hybrid filling mode (Fig. S1 of the supporting information) as described in detail elsewhere (Burian *et al.*, 2020 ▸). The single-bunch current (2 GeV electron energy) was set to 1 mA, resulting in an X-ray pulse (8 keV energy) of approximately 100 ps full width at half-maximum (FWHM). Both the laser source (PHAROS, Light Conversion, Lithuania) and the X-ray detector (EIGER2, Dectris, Switzerland), for which technical details are published by Donath *et al.* (2023 ▸), were synchronized to the storage ring time-base as depicted in Fig. 1 ▸. A digital delay generator (P400, Highland-Electronics, USA) operated at one-fourth of the ring clock frequency (289.4 kHz) and triggered the detector in order to select the single bunch. Temporal synchronization of the laser with the storage ring was achieved using the phase-comparison system (PhaseLock, TEM-Messtechnik, Germany), triggering the laser at one-eighth of the ring clock frequency (144.7 kHz). In this configuration the laser repetition rate was set to half that of the detector so that every second counted X-ray probe pulse was synchronized with the laser pump pulse [Fig. 1 ▸(*b*)], which was recorded as the pumped image with the counter A. Assuming that the SL structure is again in the steady state after 3.46 µs (verified with a long scan; data not shown), as the background image, the unpumped image was recorded with the counter B shifted by a half-period of the reference storage ring time frame. This period also corresponds to the maximum delay time between the pump and the probe signal. The X-ray detector was operated in 16-bit mode so that 65 536 X-ray pulses were recorded for every counter at a fixed delay time. The frequency of the trigger pulse train that is sent to the detector is doubled compared with the laser repetition rate. The laser wavelength was tuned to 515 nm (second harmonic of the Yb:KGW laser, 1030 nm centre wavelength, 250 fs pulse width). The energy per pulse was set to 6 µJ while the laser beam diameter was set to 0.5 mm, resulting in a fluence of 3 mJ cm^−2^ at the sample plane. Note the laser beam stabilization system (MRC, Germany) has been used to stabilize the position with an accuracy of 99.8%. The X-ray beam size was set to 0.35 × 0.1 mm (H × V), which results in an almost square footprint in the θ/2θ configuration at the scattering vector value *q* = 22.2 ± 0.8 nm^−1^ [Fig. 2 ▸(*a*)]. No laser or X-ray radiation damage was observed. The details of spatial overlap of the laser and X-ray beams are given by Burian *et al.* (2020 ▸). The data integration was performed using the open software *SAXSDOG* for real-time azimuthal integration of 2D scattering images (Burian *et al.*, 2022 ▸). The data reduction and processing was performed using *IGOR Pro* (IGOR Pro 7.0.8.1, WaveMetrics). The error propagation was calculated using the formalism described by Burian *et al.* (2020 ▸). The theoretical modelling of pump-induced transient heat transfer was performed using the *udkm1Dsim* toolbox (Schick *et al.*, 2014 ▸).

## 3. Results

### 3.1. Sample characterization

The GaAs/AlAs SL structure was grown on a GaAs(001) substrate using the molecular beam epitaxy technique. Such structures exhibit a small lattice mismatch (∼0.2%) with interfacial mixing of less than ∼3 monolayers (Robb & Craven, 2008 ▸; Cheaito *et al.*, 2018 ▸; Luckyanova *et al.*, 2018 ▸). The total thickness of the SL was designed to be 535 nm, (4.5 nm GaAs + 6.2 nm A lAs) × 50. The periodicity and crystalline structure of the SL were verified by X-ray diffraction and scanning electron microscopy (SEM) as depicted in Figs. 2 ▸(*a*) and 2(*b*), respectively. A θ–2θ rocking-curve scan shows outstanding agreement of the real SL structure with simulations performed using dynamical scattering theory (Schick *et al.*, 2014 ▸). The GaAs substrate diffraction peak at ambient temperature (300 K) was identified at the scattering vector magnitude *q* = 22.23 nm^−1^. It corresponds to the 002 reflection of the zinc blend GaAs crystal structure with the lattice constant 0.5653 nm (Adachi, 1985 ▸). The intense peaks that arise from the secondary interference of diffracted X-rays on SL periodicity represent the 0th and ±1st SL diffraction orders as highlighted in Fig. 2 ▸(*a*). The evaluated SL period is 10.76 nm (4.53 nm of GaAs/6.23 nm of AlAs), resulting in a total SL thickness of 538 nm that agrees with SEM measurements [Fig. 2 ▸(*b*)]. Prior to pump–probe experiments the SL was thermalized. The downshift (Δ*q* = 0.8 × 10^−3^ nm^−1^) of the SL 0th-order diffraction peak after laser-induced thermalization implies that the averaged SL temperature has been raised to 306.7 K, which has been further used as the SL initial temperature for simulations of transient heat conduction (see Section 3.2).

### 3.2. Double-gating performance

**Figure 3:**
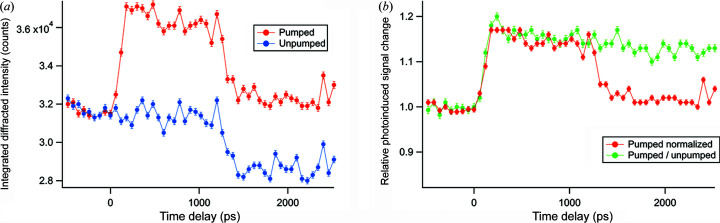
(*a*) Integrated diffracted intensity of GaAs/AlAs SL 0th-order peak in the *q* range 22.19 ± 2 × 10^−3^ nm^−1^ as a function of delay time between the laser pump and X-ray probe. (*b*) Relative photoinduced signal change in double- (green) and single-gate mode (red).

In order to obtain a temporal overlap of laser and single X-ray pulses the transient signal was measured at a fixed scattering vector magnitude of 22.19 nm^−1^ (Fig. 3 ▸), which approximately corresponds to the half-width at half-maximum offset of the GaAs/AlAs SL 0th-order peak at lower *q* [Fig. 2 ▸(*a*)]. Under such conditions the largest signal contrast is expected due to the peak broadening and its shift towards lower *q* as a consequence of pump-induced heating of the SL. In Fig. 3 ▸(*a*), counter A corresponds to the signal obtained in a single-gating experiment and counter B corresponds to the unpumped signal (background signal). The advantages of double-gate mode can be clearly seen from Fig. 3 ▸(*b*), where beam instabilities as observed in the single-gating experiment (counter A signal) vanish by simply dividing the signal of counter A with counter B, or alternatively they can be subtracted.

**Figure 4:**
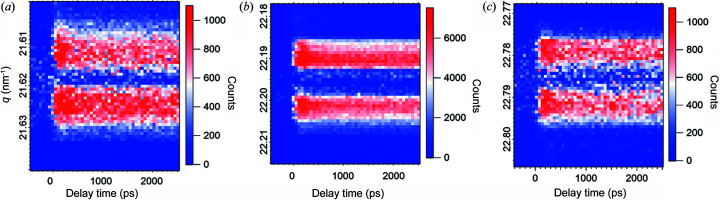
Absolute difference of transient integrated intensity changes in the proximity of (*a*) −1st, (*b*) 0th and (*c*) +1st GaAs/AlAs SL peaks. The angular increment is 0.0005° and the time delay step is 60 ps.

The absolute difference signals (|counter A − counter B|) measured at variable incidence angles, *i.e.* across the 0th and ±1st order SL peaks, as a function of delay time between the optical pump and X-ray probe, are displayed in Fig. 4 ▸. The absolute values are taken in order to highlight that transient changes induced by the pump pulse dominate due to the shift of the corresponding GaAs/AlAs SL peaks rather than their broadening [Fig. S2(*g*)]. The downshift of all three SL peaks, Δ*q* = (7 ± 0.5) × 10^−4^ nm^−1^, has been observed due to the transient SL temperature rise Δ*T* = 5.7 ± 0.4 K that corresponds to a 10 fm expansion of the single unit cell constituting the SL.

**Figure 5:**
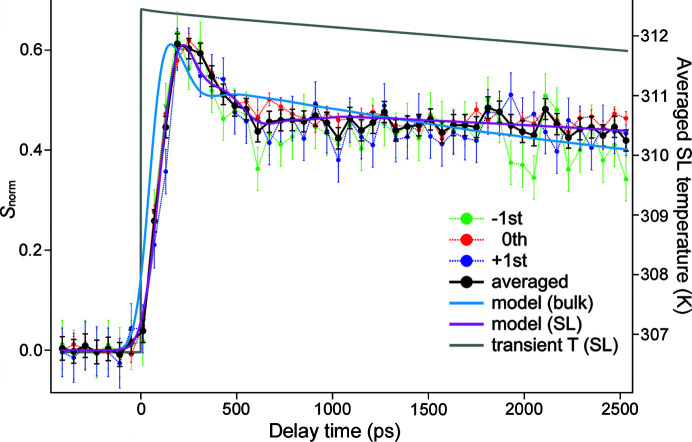
Normalized transient differential signal for −1st (green), 0th (red) and +1st (violet) GaAs/AlAs SL peaks. Their averaged trace is depicted in black. Blue and magenta solid lines represent the SL response modelled using bulk material properties and adjusted ones, respectively. The grey solid line represents the average temperature of the whole SL as function of delay time between the optical pump and X-ray probe. The displayed curves were obtained by averaging both the experimental and the theoretical data in the same 0.003° angular range as displayed in Fig. S2(*c*).

For further analysis and comparison with theoretical predictions, we nevertheless use the normalized differential signal (Fig. S2) given by

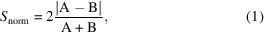

where A and B represent pumped and unpumped signals, counters A and B, respectively. Such a representation of a transient signal for the real GaAs/AlAs SL (Fig. 5 ▸) brings double the benefit. First, the calculations of transient heat transfer at a given temporal resolution can be performed for a single diffraction peak, reducing computational time (Fig. S3); and second, the further procedure of experimental data reduction for 0th and ±1st SL peaks can be easily verified. However this is not valid in general. For instance, more sophisticated structures with additional buffer layers or SLs must be carefully examined.

### 3.3. Transient heat conduction and simulations

Pump-induced transient heat conduction was modelled using the *udkm1Dsim* toolbox (Schick *et al.*, 2014 ▸). *udkm1Dsim* can simulate the structural dynamics in one-dimensional crystalline structures following an ultrashort optical excitation. It therefore allows us to evaluate cross-plane thermal conductivity, specific heat and group velocity. We found that agreement between the experimental data on a 250 ns timescale and the simulations can only be obtained with significantly reduced thermal conductivity of the GaAs/AlAs SL (Fig. S4). This is not surprising since the reduction in cross-plane thermal conductivity is a well studied phenomenon in GaAs/AlAs SL structures (Luckyanova *et al.*, 2013 ▸; Cheaito *et al.*, 2018 ▸; Giri *et al.*, 2018 ▸). Thermal transport processes at the nanoscale are complicated by the breakdown of the Fourier law of diffusion when size effects affect the mean free path of the heat carriers, which in semiconductor and dielectric materials are represented by acoustic phonons (Cahill *et al.*, 2014 ▸; Naumenko *et al.*, 2019 ▸). Different models that go beyond the diffusive regime, *e.g.* ballistic heat transport, have to be considered in order to describe such a phenomenology (Chen, 2021 ▸). Most experimental observations invoke the Casimir picture, wherein phonons travel ballistically or quasiballistically through the internal region of the sample and scatter diffusively at interfaces and boundaries (Luckyanova *et al.*, 2012 ▸; Chen, 2021 ▸). In this classical size regime, the phase information carried by phonons can be lost through diffuse scattering at boundaries and by internal scattering processes. However, if the phase information is kept, it should be possible to control the conduction of heat by manipulating phonon waves through interference effects in periodic structures and thermal band-gap formation (Maldovan, 2015 ▸). The latter is possible due to a modification of the phonon dispersion relations which control the propagation of phonons by establishing densities of modes and group velocities. In the Casimir–Knudsen regime, the local heat flux is no longer proportional to the local temperature gradient as Fourier’s law predicts but depends on the spatial temperature distribution (Chen, 2021 ▸). The *udkm1Dsim* toolbox (Schick *et al.*, 2014 ▸) provides this spatial temperature distribution, which is an alternative to solving the Boltzmann transport equations or Monte Carlo simulations with highly uncertain boundary conditions (Zeng *et al.*, 2015 ▸; Anufriev *et al.*, 2017 ▸; Cheaito *et al.*, 2018 ▸; Beardo *et al.*, 2021 ▸; Chen, 2021 ▸).

Fig. 5 ▸ shows the normalized differential GaAs/AlAs SL signal as function of delay time between the optical pump and X-ray probe. As simulations predict (Fig. S3), all three experimental curves, 0th order and two satellite SL peaks, are in good agreement The best signal-to-noise ratio under the same experimental conditions is observed for the 0th-order GaAs/AlAs SL peak due to its higher diffraction efficiency. Nevertheless, the averaged signal is used for a comparison with simulations. As for the long timescale experiment (Fig. S4), the theoretical predictions based on bulk material properties fail to describe the transient GaAs/AlAs SL response at the short (<3 ns) timescale. This is especially evident during the temperature rise and initial strain release which occur at a timescale shorter than 600 ps. At this stage the model provides a decent match only with reduced specific heat and group velocity. The heat conduction with reduced thermal conductivity effectively describes further dynamics that are valid until the SL is fully relaxed to its initial state. The adjusted GaAs/AlAs SL material properties employed for simulations and their relative decrease are summarized in Table S1 of the supporting information. On the basis of a formalism of the kinetic theory (Appendix *A* [App appa]) we also conclude that the phonon mean free path is reduced by 56%. Our current findings suggest that ultrafast experiments at sub-picosecond/picosecond timescales have to be performed in order to complete a full picture of dynamical response of optically excited GaAs/AlAs SL structures. However, for timescales above 100 ps the pump–probe setup implemented at the Austrian SAXS beamline at Elettra–Sincrotrone Trieste demonstrates a high potential, which will be enhanced by reducing the X-ray pulse width with the Elettra 2.0 upgrade (Franciosi & Kiskinova, 2023 ▸).

## 4. Conclusions

Thermal transport processes at the nanoscale are complicated by the breakdown of the Fourier law of diffusion. This is mostly due to classical size effects that lead to a significant reduction of thermal conductivity. Here, we demonstrated that, in an optically pumped 538 nm-thick GaAs/AlAs SL structure, heat conduction with reduced thermal conductivity effectively describes the structural dynamics below the 250 ns timescale. However, at short timescales (<600 ps) both reduced specific heat and group velocity determine the rise and immediate response of the SL-normalized transient differential signal. These findings have been achieved using the upgraded version of the picosecond pump–probe X-ray diffraction/scattering setup at the Austrian SAXS beamline at Elettra–Sincrotrone Trieste. We have shown that use of the double-gating mode implemented in the hybrid photon counting detector (EIGER2) helps to supress the influence of beam fluctuations and provide better data quality. The laser feedback system on the other hand kept a laser beam trajectory with 99.8% accuracy, avoiding sample temperature fluctuations. The best match between experimental and modelled data is obtained with 50 to 65% reduced specific heat, group velocity and phonon mean free path that resulted in a 12.5-fold decrease of thermal conductivity. Our findings suggest that better temporal resolution at a shorter timescale (<100 ps) is required in order to complete a full picture of dynamical response of optically excited GaAs/AlAs SL structures. Nevertheless, for timescales above 100 ps the pump–probe setup at the Austrian SAXS beamline demonstrates its high potential. A new class of experiments on bio-structures in liquid, photo-switchable systems, T-jump experiments in water *etc.* is foreseen in the near future.

## 5. Related literature

The following additional reference is cited in the supporting information: Durbin *et al.* (2012 ▸).

## Supplementary Material

Supporting information file. DOI: 10.1107/S1600576723004302/jl5061sup1.pdf

## Appendix A Kinetic theory basis

The dependence of the thermal conductivity *k* on phonon properties is given by (Chen, 2021 ▸)

where ω is the phonon angular frequency, *C*(ω) is the spectral volumetric specific heat, ν(ω) is the group velocity and Λ(ω) is the phonon mean free path. Using the relative values

(Table S1), *i.e.* the ratio of adjusted GaAs/AlAs SL material properties to volume-weighted average values, one can obtain

*i.e.* a 56% decrease in the phonon mean free path.
