# Supplementary material for: Implication of the double-gating mode in a hybrid photon counting detector for measurements of transient heat conduction in GaAs/AlAs superlattice structures
Source: J Appl Crystallogr. 2023 Jun 16;56(Pt 4):961–6. doi: 10.1107/S1600576723004302 (PMC10405594; doi:10.1107/S1600576723004302)
Supplement: Supplementary file 1 [file j-56-00961-sup1.pdf]

JOURNAL OF
APPLIED
CRYSTALLOGRAPHY

Volume 56 (2023)

Supporting information for article:

Implication of the double-gating mode in hybrid photon counting detector for measurements of transient heat conduction in GaAs/AlAs superlattice structures

Denys Naumenko, Max Burian, Benedetta Marmiroli, Richard Haider, Andrea Radeticchio, Lucas Wagner, Luca Piazza, Lisa Glatt, Stefan Brandstetter, Simone Dal Zilio, Giorgio Biasiol and Heinz Amenitsch

Supporting information

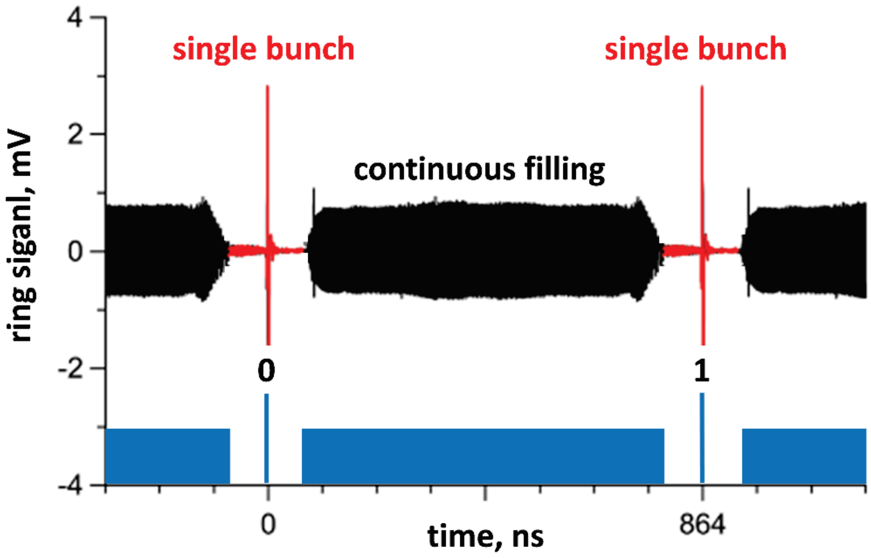

Figure S1 Ring signal of the Elettra storage ring in hybrid filling mode, consisting of continuous filling regime (black), a dark gap and a single bunch in the dark gap centre (both in red). The bottom part (blue) schematically represents a hybrid filling mode where single bunches are sequentially numbered.

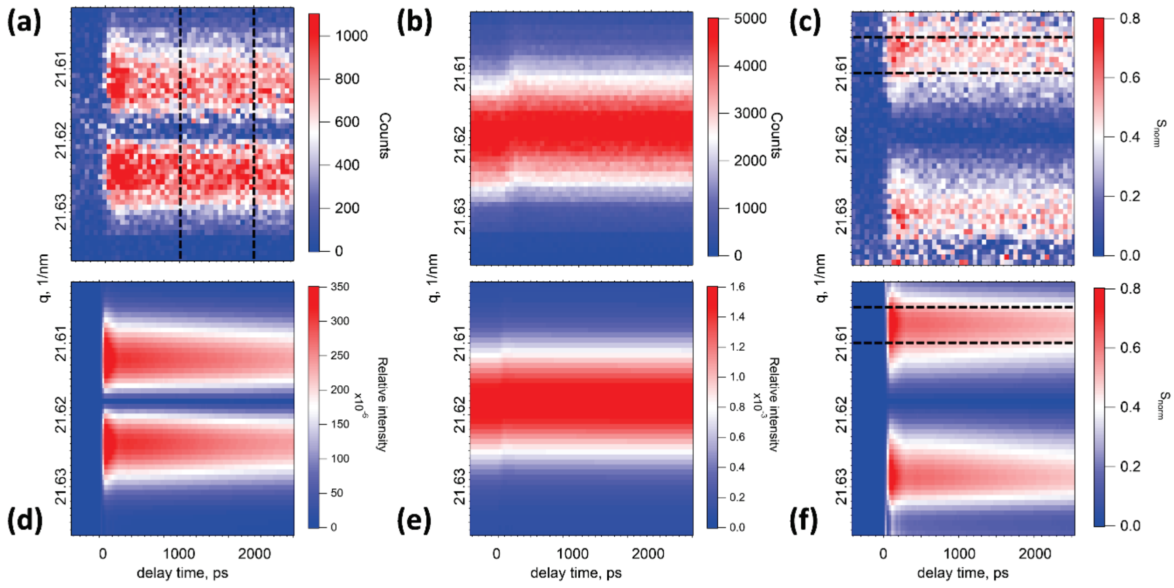

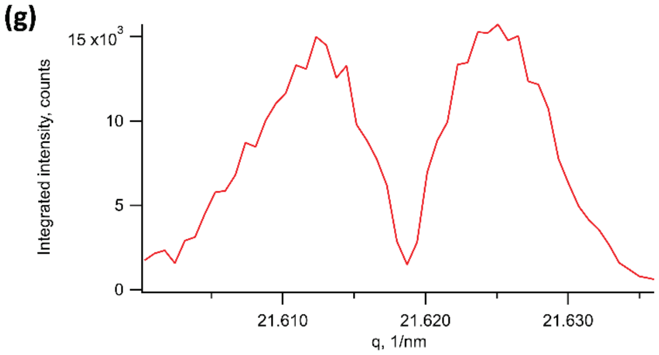

Figure S2 Experimental (a-c) and simulated (d-f) transient signals in the proximity of -1st SL peak. Panels (a, d) represent absolute difference signal; the sum is depicted in panels (b, e) while panels (c, f) show normalized differential signal given by Eq. 1 in the main text. The angular increment is 0.0005°, the time delay step is 60 ps and 30 ps for experimental (a-c) and simulated (d-f) maps, respectively. The black vertical dashed lines in panel (a) represent time window from 1000 ps to 2000 ps in which the signal has been integrated and plotted in panel (g) as function of scattering vector *q* testifying that the peak broadening is negligible. The black horizontal dashed lines in panels (c, f) represent the angular regions of 0.003° in which the signal (S_{norm}) has been averaged and plotted in Figure 5 in the main text.

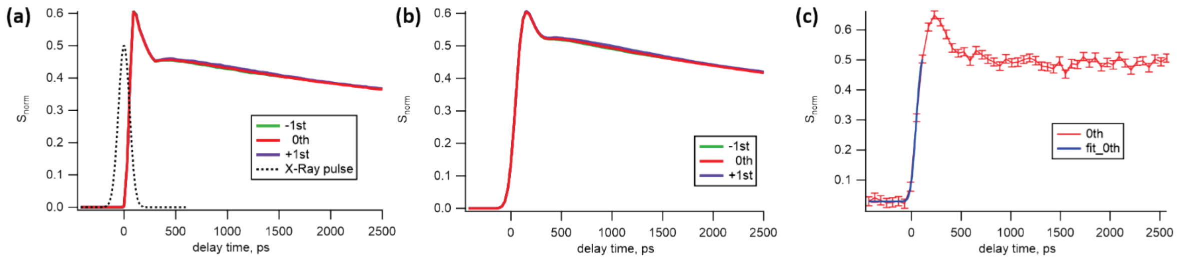

Figure S3 Simulated (a) and convoluted (b) transient response of 0th and ±1st SL peaks as function of delay time between optical pump and X-Ray probe. The X-Ray pulse width (104.6 ± 5.5 ps FWHM) has been determined from an error-function fit (Durbin *et al.,* 2012) of 0th order SL peak (c) and depicted with black dotted line in panel (a). Note that the fit range in (c) is determined by the 0th order peak position in panel (a), i.e. at 90 ps delay time.

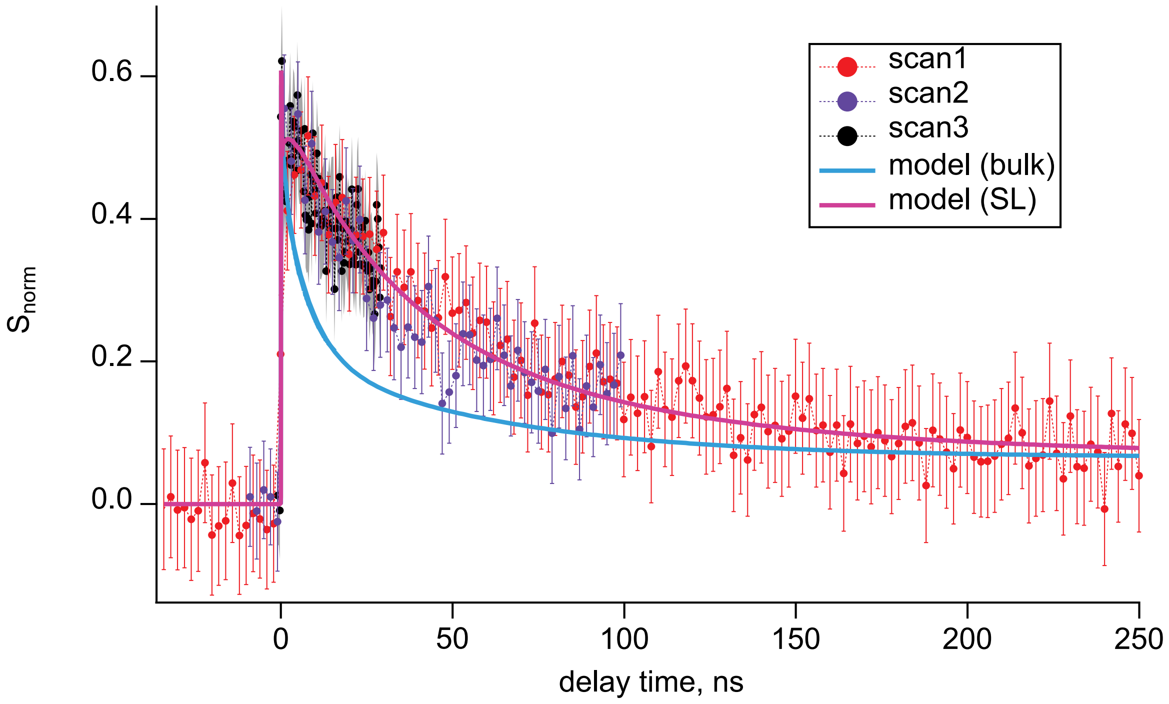

Figure S4 Normalized differential transient signals for 0th GaAs/AlAs SL peak measured in different time intervals with a variable time step (scan1-scan3). Blue and magenta solid lines represent the SL response modelled using bulk material properties and adjusted ones, respectively (see Table S1). Note that the thermal conductivity of 0.08 W/(m K) is in a good agreement with the data obtained by another methods (Luckyanova *et al.,* 2013, Cheaito *et al.,* 2018).

Table S1 Bulk GaAs, AlAs, volume weighted average (GaAs/AlAs *) and adjusted GaAs/AlAs SL material properties employed for simulations as well as their relative changes $\frac{\text{GaAs/AlAs SL}}{\text{GaAs/AlAs}^*}$.

	GaAs[a]	AlAs[a]	GaAs/AlAs *	GaAs/AlAs SL	$\frac{\text{GaAs/AlAs SL}}{\text{GaAs/AlAs}^*}$
Lattice constant, Å	5.653	5.661	5.658 *	5.658 *	1
Thermal expansion coefficient, ($x10^{-6}$ K)	6.4	5.2	5.7 *	5.7 *	1
Specific heat, J/(kg K)	330	450	400	150	0.38
Sound velocity, km/s	4.73	5.65	5.26	2.50	0.48
Thermal conductivity, W/(m K)	55	91	76	6	0.08

[a] (Adachi, 1985).

* volume weighted average.

References

Adachi, S. (1985). *Journal of Applied Physics*, *58*(3), R1–R29.

Cheaito, R., Polanco, C. A., Addamane, S., Zhang, J., Ghosh, A. W., Balakrishnan, G., & Hopkins, P. E. (2018). *Physical Review B*, *97*(8), 085306.

Durbin, S. M., Clevenger, T., Graber, T. & Henning, R. (2012). Nature Photonics, 6, 111–114.

Luckyanova, M. N., Johnson, J. A., Maznev, A. A., Garg, J., Jandl, A., Bulsara, M. T., Fitzgerald, E. A., Nelson, K. A., & Chen, G. (2013). *Nano Letters*, *13*(9), 3973–3977.
